# COVID-19 Vaccine Hesitancy Among Health Care Professionals of Allied Hospitals of Rawalpindi Medical University: A Mediation Analysis

**DOI:** 10.1192/bjo.2022.239

**Published:** 2022-06-20

**Authors:** Sajeel Saeed, Abdul Rauf, Waqar Younas, Muhammad Arish, Abdul Sannan

**Affiliations:** Rawalpindi Medical University, Rawalpindi, Pakistan

## Abstract

**Aims:**

This research assessed healthcare workers' vaccination practices for influenza, hepatitis, and pneumonia, as well as their desire to get COVID-19 vaccine when accessible, and investigated their 7C psychological antecedents (confidence, complacency, collective responsibility, compliance, calculation, constraints and conspiracy). Stress variables and vaccination intention for COVID-19 were also compared to see whether psychological stress had a meditative impact on the relationship.

**Methods:**

An analytical cross-sectional survey was conducted among health care professionals including nurses and doctors of tertiary care hospitals of Rawalpindi. Data were collected from February to April 2021 to get the COVID-19 vaccination when it became available, and looked into their 7C mental forebears (confidence, complacency, collective responsibility, compliance, calculation, constraints and conspiracy). A total of 642 health care professionals voluntarily participated in our research. Demographic Details, questions like pneumonia vaccination, hepatitis vaccination, influenza vaccination and 7C questions were asked. Data were entered and analysed through SPSS 26.0 and Python. Correlation, linear and non linear regression, and mediation analysis were applied.

**Results:**

The immunization rates for influenza, hepatitis, and pneumonia vaccines, as well as the percentage of those who received COVID-19 vaccination, were 43.4%, 65.2%, 42.8%, and 39.7%, respectively. Hepatitis vaccination was significantly associated with the 7C model, influenza vaccination with conspiracy, whereas pneumonia was significantly associated with compliance. confidence (r = 0.11), complacency (r = −0.19), constraints (r = −0.20), calculation (r = 0.08), collective responsibility (r = 0.18), and compliance (r = 0.19) were significantly corelated with the COVID-19 Vaccine Intention. Contrary to direct effect, the indirect effect of patient contact frequency (β = −0.01, ρ<0.05) and terrified of contracting virus (β = −0.05, ρ<0.05) via psychological stress on COVID-19 Vaccination intention were significant depicting full mediation.

**Conclusion:**

For herd immunity, the probability for COVID-19 vaccination uptake among healthcare professionals was less than optimal. The 7C structures may help predict immunization against influenza, hepatitis, and pneumonia, but not vaccination against COVID-19.

